# Iron‐Dependent JMJD1A‐Mediated Demethylation of H3K9me2 Regulates Gene Expression During Adipogenesis in a Spatial Genome Organization‐Dependent Manner

**DOI:** 10.1111/gtc.70023

**Published:** 2025-04-28

**Authors:** Shinnosuke Masuda, Tetsuro Komatsu, Safiya Atia, Tomohiro Suzuki, Mayuko Hayashi, Atsushi Toyoda, Hiroshi Kimura, Takeshi Inagaki

**Affiliations:** ^1^ Laboratory of Epigenetics and Metabolism Institute for Molecular and Cellular Regulation, Gunma University Maebashi Gunma Japan; ^2^ Advanced Genomics Center National Institute of Genetics Mishima Japan; ^3^ Cell Biology Center Institute of Integrated Research, Institute of Science Tokyo Yokohama Japan

**Keywords:** A/B compartments, adipocyte differentiation, H3K9me2, histone demethylase, iron‐dependent enzyme activity, JMJD1A, lamina‐associated domains

## Abstract

Chromatin restructuring across multiple hierarchical scales directs lineage‐specific gene expression during cell differentiation. Here, we investigated the iron‐dependent demethylation of histone H3 lysine 9 dimethylation (H3K9me2) by the demethylase jumonji domain‐containing 1A (JMJD1A) in adipocyte differentiation. Using the 3T3‐L1 adipocyte differentiation model, we show that JMJD1A knockdown increases H3K9me2 levels, whereas forced expression of wild‐type JMJD1A reduces H3K9me2 levels within the A compartment, as defined by megabase scale high‐throughput chromosome conformation capture (Hi‐C) data. In contrast, a JMJD1A mutant defective in iron coordination was unable to demethylate H3K9me2. Genome‐wide analyses of H3K9me2 levels at transcription start sites on a kilobase scale demonstrated that JMJD1A targets genes involved in peroxisome proliferator‐activated receptor signaling and lipid metabolism in an iron‐dependent manner, supporting a model in which H3K9me2 demethylation facilitates adipogenic transcription networks. Furthermore, we examined the relationship between H3K9me2 and lamin B1 levels within lamina‐associated domains (LADs) specifically reorganized during differentiation. Although LADs with higher H3K9me2 exhibited modestly elevated lamin B1 association in preadipocytes, lamin B1 levels declined during differentiation regardless of H3K9me2 status. These findings emphasize the importance of the iron‐dependent enzymatic function in JMJD1A and broaden our understanding of how specific H3K9 demethylases coordinate compartmentalized epigenetic programs during adipogenesis.

## Graphical abstract

JMJD1A‐mediated H3K9me2 demethylation is primarily confined to A compartments on a megabase scale during adipogenesis. Genome‐wide H3K9me2 analysis on a kilobase scale demonstrated that JMJD1A iron‐dependently targets genes involved in PPAR signaling and lipid metabolism.

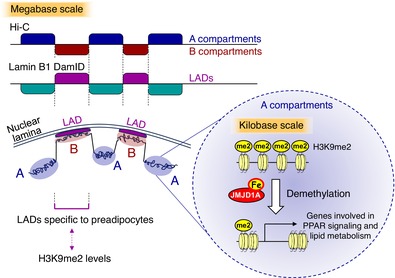

## 1 Introduction

In the regulation of cell differentiation, genes specific to a given lineage are activated through controlled structural changes in chromatin, whereas genes specific to alternative lineages are suppressed. It has become clear that the controlled structural changes in chromatin underlying such differentiation processes operate at multiple hierarchical levels: large‐scale structural changes on the megabase scale, physical interactions through chromatin loops at the sub‐megabase scale, and more localized mechanisms at the kilobase scale. For example, in terms of megabase scale structural changes, undifferentiated pluripotent cells demonstrate a generally relaxed chromatin architecture, whereas more lineage‐committed cells show overall chromatin condensation (Chen and Dent 2014). At the sub‐megabase scale (usually 500 kb–1 Mb), topologically associating domains (TADs) are observed. These domains show a high degree of conservation in each cell type and frequently undergo structural changes such as physical interactions through chromatin loops within each domain (da Costa‐Nunes and Noordermeer 2023). Moreover, at a more localized, kilobase‐scale level, cells with relatively open chromatin, such as undifferentiated pluripotent cells, are known to establish a “bivalent” chromatin modification consisting of the transcription‐activating mark histone H3 lysine 4 trimethylation (H3K4me3) and the transcription‐repressing mark histone H3 lysine 27 trimethylation (H3K27me3), thereby repressing lineage‐specific gene expression (Chen and Dent 2014). In preadipocytes, which are along the lineage toward adipocytes, there is also a reported transcriptional repression mechanism, centered on a bivalent mark involving H3K9 and H3K4 methylation, which contributes to maintaining their undifferentiated state (Matsumura et al. 2015).

In large‐scale chromatin structures spanning broad regions, domains that are globally condensed often appear as regions of high electron density at the nuclear periphery by electron microscope and are referred to as heterochromatin (Allshire and Madhani 2018). Compared with more open euchromatin, heterochromatin is associated with transcriptional silencing and increases upon cell differentiation relative to pluripotent cells (Chen and Dent 2014). Hi‐C analyses demonstrated that euchromatin and heterochromatin form active (A) and inactive (B) compartments, respectively, which correlate with the activation or suppression of gene expression (Lieberman‐Aiden et al. 2009). The B compartment is linked to LADs, which interact with the nuclear lamina. Epigenetically, the B compartment is characterized by abundant H3K9 methylation, which is considered to repress transcription by controlling chromatin condensation and restricting transcription factor access (Padeken et al. 2022). Thus, H3K9 methylation plays an important role in forming heterochromatin and the B compartment, mediating widespread transcriptional repression and chromatin condensation. On the other hand, H3K9 methylation is not exclusively found in the B compartment and is also known to function within euchromatic or A compartment regions, where it has been shown to mediate selective and dynamic gene repression, particularly at kilobase‐scale regions as well as on a larger scale (Yan et al. 2020; Smith et al. 2021; Padeken et al. 2022). Overall, H3K9 methylation occurs in both the A and B compartments, but its regulatory profile differs between these compartments.

A recent study using embryonic stem (ES) cells demonstrated that H3K9 methylation is regulated in a compartment‐specific manner by different H3K9 methyltransferases in the A and B compartments. Among the five H3K9 methyltransferases (SUV39H1, SUV39H2, SETDB1, G9a [also known as EHMT2], and G9a‐like protein [GLP, also known as EHMT1]), the heterodimer G9a/GLP, together with SETDB1, regulates H3K9me2 in both the A and B compartments, whereas SUV39H1 and SUV39H2 methylate H3K9me2 exclusively in the B compartment (Fukuda et al. 2021). However, in the context of H3K9me2 demethylation, it remains unclear how H3K9me2 demethylases are differentially utilized between the A and B compartments. In this study, we used an adipocyte differentiation model to investigate how demethylation of H3K9 by the H3K9 demethylase JMJD1A (also known as KDM3A and JHDM2A) is distributed between the A and B compartments. In the 3T3‐L1 adipocyte differentiation model, differentiation is induced over 8–9 days by treating post‐confluent cells with a differentiation cocktail containing insulin, dexamethasone, and 3‐isobutyl‐1‐methylxanthine (IBMX). During this process, the binding of various transcription factors, along with epigenetic modifications in target genomic regions, coordinates to drive differentiation. Among these transcription factors, peroxisome proliferator‐activated receptor gamma (PPARγ) is a well‐established master regulator of adipocyte differentiation (Farmer 2006; Inagaki et al. 2016). Epigenetically, a bivalent mechanism involving H3K9 and H3K4 methylation has been reported to repress the transcription of loci, such as *Pparg*, which encodes PPARγ, thereby contributing to the maintenance of the undifferentiated state in 3T3‐L1 preadipocytes (Matsumura et al. 2015). In addition, we recently found that the H3K9me2 demethylase JMJD1A removes H3K9me2 marks around the *Pparg* locus, thereby promoting *Pparg* expression and inducing 3T3‐L1 cell differentiation in an iron‐dependent manner during the early stage of differentiation (day 2) (Suzuki et al. 2023). As PPARγ itself is a transcription factor, the JMJD1A‐dependent upregulation of *Pparg* can directly affect the transcription of other genes in pre‐existing open chromatin regions, independent of H3K9 demethylation. At the same time, H3K9me2 demethylation by JMJD1A relaxes the chromatin structure; therefore, newly synthesized PPARγ can also bind these demethylated open chromatin regions to regulate a wide range of genes in coordination with JMJD1A.

Based on these considerations, we aimed to identify the target genes that are iron‐dependently regulated by JMJD1A through H3K9me2 demethylation and to elucidate the pathways controlling their expression during adipocyte differentiation of 3T3‐L1 preadipocytes. Notably, TAD boundaries are reported to remain largely unchanged during 3T3‐L1 adipocyte differentiation at a 50‐kb resolution (Siersbæk et al. 2017), implying that transcriptional regulation in adipogenesis occurs primarily at scales below 50 kb within A and B compartments. In the present study, we identified JMJD1A target genes that are subject to H3K9me2‐dependent regulation and investigated the differential regulation of JMJD1A‐driven H3K9me2 demethylation in A and B compartments.

## 2 Results

### 2.1 JMJD1A‐Mediated H3K9 Demethylation During Adipocyte Differentiation Aligns With the A Compartment at the Megabase Scale

**FIGURE 1:**
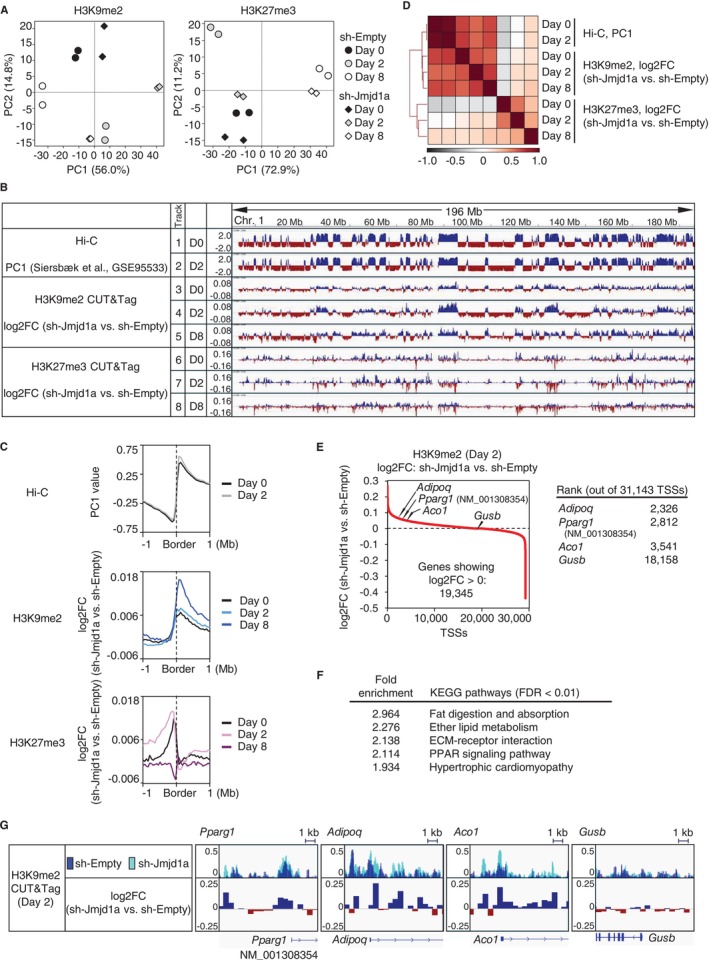
JMJD1A‐Mediated H3K9me2 Demethylation Associates with the A Compartment at the Megabase Scale, and with Adipocyte Gene TSSs at the Kilobase Scale. (A) PCA plots of H3K9me2 (left panel) and H3K27me3 (right panel). CUT&Tag datasets obtained from JMJD1A‐KD (sh‐Jmjd1a) and control (sh‐Empty) 3 T3‐L1 cells at days 0, 2, and 8 of differentiation are shown, with duplicate samples for each condition. (B) Comparison of changes in histone modifications upon JMJD1A‐KD in the A compartment and the B compartment. The log_2_ fold changes (log2FC) in H3K9me2 (Tracks 3–5) and H3K27me3 (Tracks 6–8) in JMJD1A‐KD (sh‐Jmjd1a) cells relative to control (sh‐Empty) cells at days 0, 2, and 8 of differentiation are shown alongside published Hi‐C data (GSE95533), where PC1 values on days 0 and 2 of differentiation are depicted in Tracks 1 and 2. The entire region of chromosome 1 is shown in a genome browser view. D0, D2, and D8 indicate days 0, 2, and 8 of differentiation, respectively. (C) Aggregation plots of Hi‐C PC1 (top panel), H3K9me2 (middle panel), and H3K27me3 (bottom panel) around the A/B compartment boundaries. The boundaries were determined based on the day 2 Hi‐C PC1 values. Data from days 0, 2, and 8 of differentiation are shown within ±1 Mb of the boundaries. (D) Pearson correlation matrix of Hi‐C and CUT&Tag datasets. The matrix was calculated in 100‐kb bins across the genome to illustrate the correlations among H3K9me2, H3K27me3, and Hi‐C data. (E) and (F) H3K9me2 changes around TSSs and KEGG pathway analysis. In (E), the 31,143 TSS regions (±1.5 kb) on day 2 of differentiation were ranked by descending order of their H3K9me2 log2FC in JMJD1A‐KD (sh‐Jmjd1a) cells relative to control (sh‐Empty) cells (left panel). Among these regions, 19,345 TSS regions had positive log2FC values. The ranks of the adipocyte‐associated genes (*Adipoq*, *Pparg1*, and *Aco1*) and the housekeeping gene (*Gusb*) are highlighted (right panel). In (F), the top 5000 TSS regions demonstrating the largest H3K9me2 increases (i.e., the highest positive log2FC) in JMJD1A‐KD (sh‐Jmjd1a) cells relative to control (sh‐Empty) cells were annotated to their nearest genes. KEGG pathway analysis of these annotated genes with FDR < 0.01 is presented in descending order of fold enrichment. (G) Genome browser tracks of representative adipocyte‐associated genes and a housekeeping gene (*Gusb*). The upper panel shows overlaid H3K9me2 CUT&Tag signals (50‐bp bin size) in JMJD1A‐KD (sh‐Jmjd1a) cells and control (sh‐Empty) cells for *Adipoq*, *Pparg1*, *Aco1*, and *Gusb*. The lower panel shows the H3K9me2 log2FCs (sh‐Jmjd1a vs. sh‐Empty) in 500‐bp bins for the same genes.

To investigate the regulatory role of JMJD1A in adipocyte differentiation via the demethylation of histone H3K9me2, we analyzed genome‐wide changes in H3K9me2 demethylation. Using JMJD1A knockdown (JMJD1A‐KD) 3T3‐L1 cells and control (sh‐Empty) 3T3‐L1 cells, established by infecting 3T3‐L1 cells with a retrovirus expressing short hairpin RNA (shRNA) targeting *Jmjd1a* (sh‐Jmjd1a) or a retrovirus not expressing shRNA (sh‐Empty), respectively, followed by antibiotic selection (Suzuki et al. 2023), we performed cleavage under targets and tagmentation (CUT&Tag) assays using an anti‐H3K9me2 antibody on days 0, 2, and 8 of adipocyte differentiation (Figure 1A, left and Figure S1). To evaluate H3K9me2 changes across the genome at the megabase scale, we compared H3K9me2 methylation levels in JMJD1A‐KD cells with those in control (sh‐Empty) cells, representing the differences as log_2_ fold change (log2FC) (Figure 1B). We observed that H3K9me2 levels were not uniformly increased across all genomic regions in JMJD1A‐KD cells (Figure 1B); instead, increases occurred in a region‐specific manner. Further comparison of these results with existing Hi‐C data on higher‐order chromatin architecture in 3T3‐L1 cells (GSE95533) (Siersbæk et al. 2017) demonstrated that JMJD1A‐dependent H3K9me2 demethylation predominantly aligns with the A compartment, characterized by positive values of the first principal component (PC1) from Hi‐C analysis at the megabase scale (Figure 1B). This alignment was further corroborated by the observation that the boundary where H3K9me2 levels shifted from negative to positive log2FC in JMJD1A‐KD (sh‐Jmjd1a) cells compared to control (sh‐Empty) cells coincided with the boundary between the B and A compartments (Figure 1C).

To determine whether a similar trend occurs for another repressive histone mark, H3K27me3, we performed CUT&Tag with an anti‐H3K27me3 antibody. However, no comparable trend aligned with the A compartment (Figure 1B–D). Thus, at the megabase scale, JMJD1A‐mediated H3K9me2 demethylation appears to be confined to the A compartment during adipocyte differentiation.

As that H3K9 methylation within the A compartment is associated with selective and dynamic gene repression, and promoter‐anchored chromatin looping within TADs is crucial for adipocyte differentiation (Siersbæk et al. 2017; Inagaki 2018), we hypothesized that JMJD1A modulates transcription by regulating the chromatin structure of discrete gene loci within the A compartment.

### 2.2 JMJD1A‐Dependent H3K9me2 Demethylation at the Transcription Start Site (TSS) Regions of Adipocyte‐Associated Genes

Next, to further clarify how JMJD1A might regulate individual loci through H3K9me2 demethylation, we conducted a genome‐wide analysis focused on the TSS regions (TSS ± 1.5 kb) of each gene. Specifically, we analyzed H3K9me2 levels in JMJD1A‐KD cells compared with control cells on day 2 of adipocyte differentiation, calculated the log2FC values, and then ranked all TSS regions by these values. Among the 31,143 TSS regions analyzed, 19,345 demonstrated a positive H3K9me2 fold change upon *Jmjd1a* KD (Figure 1E). These TSS regions were matched to their respective genes, and the Kyoto Encyclopedia of Genes and Genomes (KEGG) pathway analysis was performed on the top 5000 TSSs ranked by log2FC. The analysis demonstrated significant enrichment in pathways such as “Fat digestion and absorption”, “Ether lipid metabolism,” “ECM receptor interaction,” and the “PPAR signaling pathway” (Figure 1F). These results suggest that JMJD1A plays a key role in regulating lipid metabolism and PPARγ signaling.

Notably, this set of genes included *Pparg1* (specifically the NM_001308354 variant), which we previously reported to be regulated by JMJD1A during adipocyte differentiation, as well as *Adipoq* and *Aco1*, which are genes closely associated with adipocyte biogenesis and lipid metabolism, respectively (Figure 1E). At these loci, a kilobase‐scale analysis confirmed that H3K9me2 levels were higher around the TSS in JMJD1A‐KD cells relative to controls, in contrast to housekeeping genes such as *Gusb*, which did not demonstrate similar changes (Figure 1G).

### 2.3 Iron‐Dependent JMJD1A Activity Regulates H3K9me2 in the Regions of Adipogenesis‐Related Genes

**FIGURE 2:**
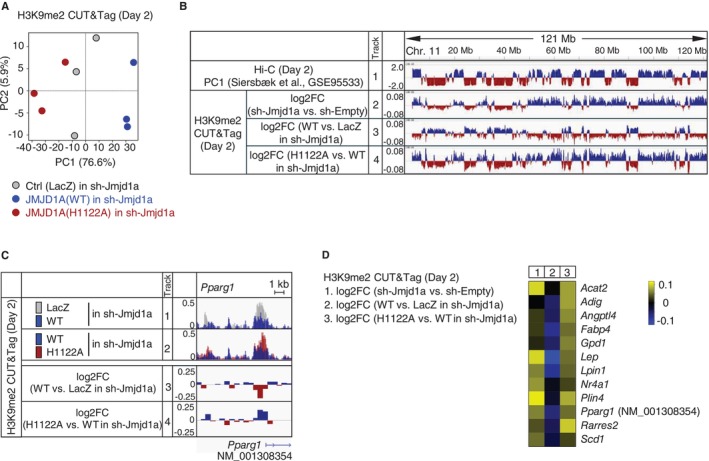
Iron‐Dependent JMJD1A‐Mediated H3K9me2 Demethylation is Associated with the A Compartment at the Megabase Scale and with Adipocyte Gene TSSs at the Kilobase Scale. (A) PCA plots of H3K9me2 CUT&Tag datasets in JMJD1A‐KD (sh‐Jmjd1a) cells overexpressing either LacZ (control), JMJD1A(WT), or JMJD1A(H1122A) on day 2 of differentiation, with triplicate samples for each condition. (B) Genome browser view of H3K9me2 modifications in rescue experiments, and their association with A/B compartments. The log2FC of H3K9me2 on day 2 of differentiation is shown for three comparisons: JMJD1A‐KD (sh‐Jmjd1a) cells compared with control (sh‐Empty) cells (Track 2), JMJD1A(WT)‐expressing JMJD1A‐KD (sh‐Jmjd1a) cells compared with JMJD1A‐KD (sh‐Jmjd1a) cells expressing the LacZ control (Track 3), and JMJD1A(WT)‐expressing JMJD1A‐KD (sh‐Jmjd1a) cells compared with JMJD1A(H1122A)‐expressing JMJD1A‐KD (sh‐Jmjd1a) cells (Track 4). Published Hi‐C PC1 values for chromosome 11 (GSE95533) are shown in Track 1. The entire chromosome 11 region is shown in the genome browser view. (C) Genome browser view of the *Pparg1* locus. Tracks 1 and 2 show overlaid H3K9me2 signals (50‐bp bins) for comparisons between JMJD1A(WT)‐expressing JMJD1A‐KD (sh‐Jmjd1a) cells and LacZ control JMJD1A‐KD (sh‐Jmjd1a) cells (Track 1), as well as JMJD1A(WT)‐expressing JMJD1A‐KD (sh‐Jmjd1a) cells and JMJD1A(H1122A)‐expressing JMJD1A‐KD (sh‐Jmjd1a) cells (Track 2). Tracks 3 and 4 (500‐bp bins) show the corresponding log2FC values for the same comparisons. (D) Heatmap showing H3K9me2 log2FC around TSSs (±1.5 kb) for comparisons on day 2 of differentiation. Column 1 shows a comparison between JMJD1A‐KD (sh‐Jmjd1a) cells and control (sh‐Empty) cells. Column 2 represents a comparison between JMJD1A(WT)‐expressing JMJD1A‐KD (sh‐Jmjd1a) cells and JMJD1A‐KD (sh‐Jmjd1a) cells expressing the LacZ control. Column 3 shows a comparison between JMJD1A(WT)‐expressing JMJD1A‐KD (sh‐Jmjd1a) cells and JMJD1A(H1122A)‐expressing JMJD1A‐KD (sh‐Jmjd1a) cells. The genes shown are those belonging to GO: 0045444 (Fat cell differentiation) and KEGG pathways (PPAR signaling pathway, Fat digestion and absorption, and Glycerophospholipid metabolism).

Iron‐dependent enzymatic function is extremely important for JMJD1A‐mediated regulation of *Pparg* in white adipocytes (Suzuki et al. 2023). However, whether JMJD1A also regulates H3K9 demethylation at loci other than *Pparg* in an iron‐dependent manner remains unclear. To address this, we conducted a genome‐wide analysis of iron‐dependent JMJD1A‐mediated H3K9me2 demethylation in adipocytes. To this end, we utilized previously reported cell lines, including JMJD1A‐KD cells overexpressing either a wild‐type JMJD1A [JMJD1A(WT)], an iron‐coordination‐site defective mutant [JMJD1A(H1122A)] (Nakamura et al. 2012; Schneider et al. 2015), or a lacZ reporter gene encoding cytoplasmic β‐galactosidase, established by retroviral infection followed by antibiotic selection (Suzuki et al. 2023). Both JMJD1A(WT) and JMJD1A(H1122A) were engineered to have shRNA‐resistant mutations to prevent interference from shRNA targeting *Jmjd1a*. CUT&Tag analysis using an anti‐H3K9me2 antibody was performed on each cell line on day 2 of adipocyte differentiation. Principal component analysis (PCA) demonstrated clear separation among the groups (*n* = 3; Figure 2A). At the megabase scale, enforced expression of JMJD1A(WT) in the *Jmjd1a* KD background resulted in lower H3K9me2 levels compared with enforced expression of a lacZ control (i.e., low log2FC) in A compartment regions (Figure 2B, track 3). In contrast, cells expressing the iron‐coordination‐site defective JMJD1A (H1122A) demonstrated relatively higher H3K9me2 levels (i.e., higher log2FC) in these same regions (Figure 2B, track 4). These observations indicate that, at the megabase scale, JMJD1A‐mediated H3K9me2 demethylation during adipocyte differentiation occurs primarily in the A compartment.

We next analyzed changes at the kilobase scale within discrete gene regions in the A compartment, focusing first on the *Pparg1* locus (NM_001308354). In the TSS region of *Pparg1*, enforced expression of JMJD1A(WT) restored H3K9 demethylation in JMJD1A‐KD cells, whereas this effect was not detected in cells expressing JMJD1A (H1122A) (Figure 2C). In addition to *Pparg1*, similar trends were observed in other adipogenesis‐associated genes, including *Acat2*, *Adig*, *Angptl4*, *Fabp4*, *Gpd1*, *Lep*, *Lpin1*, *Nr4a1*, *Plin4*, *Rarres2*, and *Scd1* (Figure 2D). These genes fall under the Gene Ontology (GO) terms associated with fat differentiation (*Adig*, *Fabp4*, *Lep*, *Lpin1*, *Nr4a1*, *Plin4*, *Rarres2*, and *Scd1*) or are categorized within the KEGG pathways such as “PPAR signaling” (*Angptl4*, *Plin4*), “Fat digestion and absorption” (*Acat2*), and “Glycerophospholipid metabolism” (*Gpd1*). Collectively, these findings suggest that JMJD1A, through its iron‐dependent enzymatic activity, controls H3K9me2 levels at crucial genomic loci involved in adipocyte differentiation.

### 2.4 Identification of Differentially Expressed Genes (DEGs) Under JMJD1A KD and Rescue Conditions

**FIGURE 3:**
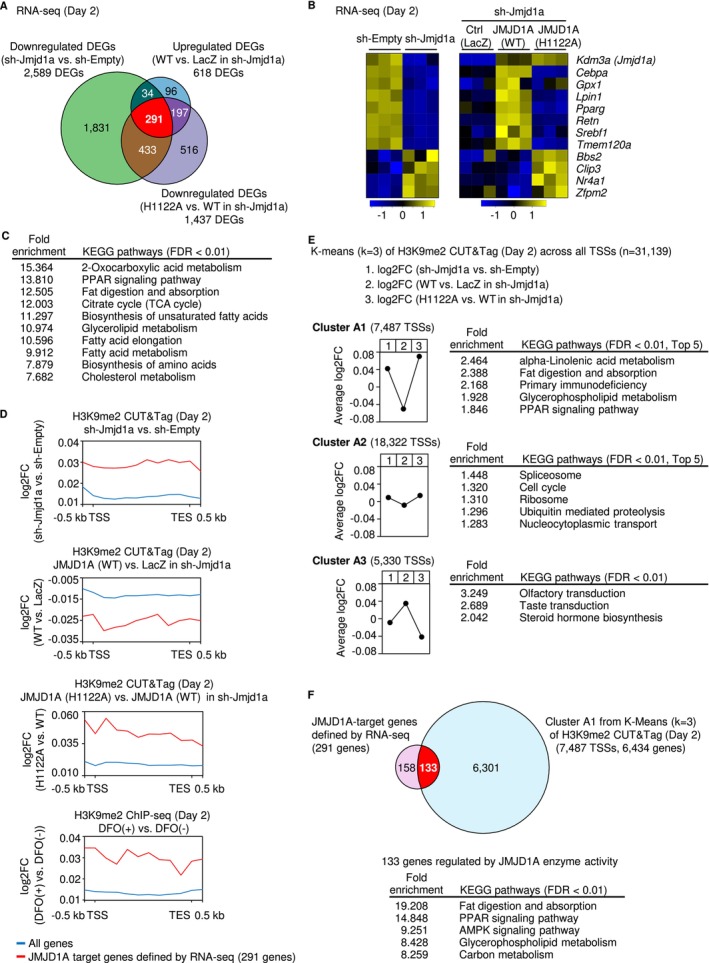
Regulation of PPARγ Signaling and Expression of Metabolic Genes by Iron‐Dependent JMJD1A‐Mediated H3K9me2 Demethylation during Adipocyte Differentiation. (A) Venn diagram of differentially expressed genes (DEGs). Three DEG sets from day 2 of differentiation are shown: Genes downregulated in JMJD1A‐KD (sh‐Jmjd1a) cells compared with control (sh‐Empty) cells, genes upregulated in JMJD1A(WT)‐expressing JMJD1A‐KD (sh‐Jmjd1a) cells compared with LacZ‐expressing JMJD1A‐KD cells, and genes downregulated in JMJD1A(H1122A)‐expressing JMJD1A‐KD cells compared with JMJD1A(WT)‐expressing JMJD1A‐KD cells. A total of 291 genes that were downregulated upon JMJD1A KD and restored by JMJD1A(WT) [but not by JMJD1A(H1122A)] were identified as JMJD1A target genes. (B) Heatmap of selected JMJD1A target genes. Genes belonging to GO: 0045444 (Fat cell differentiation), along with *Jmjd1a* itself, are shown with their relative expression (*z*‐score) on day 2 of differentiation. (C) KEGG pathway analysis of the 291 JMJD1A target genes. Using FDR < 0.01 as a cutoff, significantly enriched KEGG pathways are shown in descending order of fold enrichment. (D) Aggregation plots of H3K9me2 across gene bodies. H3K9me2 levels from the TSS to the TES ±0.5 kb are shown as log2FC for the following four comparisons (from top to bottom): JMJD1A‐KD (sh‐Jmjd1a) cells vs. control (sh‐Empty) cells, JMJD1A(WT)‐expressing JMJD1A‐KD cells versus LacZ‐expressing JMJD1A‐KD cells, JMJD1A(H1122A)‐expressing JMJD1A‐KD cells vs. JMJD1A(WT)‐expressing JMJD1A‐KD cells, and DFO‐treated vs. vehicle‐treated 3 T3‐L1 cells. Red lines represent the 291 JMJD1A target genes, whereas the blue lines represent all genes. Four genes with mismatched gene IDs between RNA‐seq and CUT&Tag datasets were excluded. (E) K‐means clustering of TSS‐centered H3K9me2 changes. For 31,139 TSS regions (±1.5 kb) on day 2 of differentiation, log2FC values from three comparisons (1, JMJD1A‐KD (shJmjd1a) cells vs. control (sh‐Empty) cells; 2, JMJD1A(WT)‐expressing JMJD1A‐KD cells vs. LacZ‐expressing JMJD1A‐KD cells; and 3, JMJD1A(H1122A)‐expressing JMJD1A‐KD cells vs. JMJD1A(WT)‐expressing JMJD1A‐KD cells) were subjected to K‐means clustering. This analysis yielded Clusters A1 (7487 TSSs), A2 (18,322 TSSs), and A3 (5330 TSSs). The average log2FC trends are plotted for each cluster. After annotating the genes in each cluster, KEGG pathway analysis (FDR < 0.01) was performed, and the top pathways by fold enrichment are listed. Cluster A1 includes genes with increased H3K9me2 upon JMJD1A KD that were demethylated by JMJD1A(WT) but not by JMJD1A(H1122A), indicating iron‐dependent regulation. (F) Identification of 133 genes regulated by iron‐dependent JMJD1A‐mediated demethylation. Venn diagram (top panel) comparing the 291 JMJD1A target genes in (A) with the 6434 genes mapped to Cluster A1 in (E). From this overlap, 133 genes were defined as those in which transcription is enhanced by iron‐dependent JMJD1A demethylation (see also Figure S2). These 133 genes were further subjected to KEGG pathway analysis (FDR < 0.01), and the significantly enriched pathways are listed in descending order of fold enrichment.

To investigate how JMJD1A affects transcription during the early stages of adipocyte differentiation, we performed RNA‐sequencing (RNA‐seq) analysis on cells on day 2 after differentiation induction. First, we compared JMJD1A‐KD (sh‐Jmjd1a) cells with control (sh‐Empty) cells using edgeR software, applying a false discovery rate (FDR) cutoff of < 0.05 to identify DEGs. As a result, 2589 DEGs were identified (Figure 3A). Next, we analyzed JMJD1A‐KD (sh‐Jmjd1a) cells complemented with JMJD1A (WT) and compared them to cells infected with a LacZ‐expressing control virus, thus identifying DEGs from complementation experiments. To further investigate the genes regulated by JMJD1A in an iron‐dependent manner, we compared cells expressing JMJD1A(WT) with those expressing the iron‐coordination‐site defective mutant JMJD1A (H1122A). An integrated analysis of these DEGs demonstrated a subset of 291 genes regulated by JMJD1A in an iron‐dependent manner (Figure 3A). This gene set included *Cebpa*, *Lpin1*, *Pparg*, *Serbf1*, *Retn*, and *Tmem120a*, all of which are categorized under the GO term 0045444 (Fat cell differentiation) (Figure 3B). In contrast, additional genes in the same GO term, namely *Bbs2*, *Clip3*, *Nr4a1*, and *Zfpm2*, demonstrated reciprocal expression changes, suggesting that some loci may respond secondarily to JMJD1A‐mediated H3K9me2 modulation (Figure 3B). Subsequent KEGG pathway analysis of these 291 iron‐dependent JMJD1A‐regulated genes showed that “PPAR signaling” was prominently enriched (fold enrichment: 13.810), consistent with its central role in adipocyte differentiation. Other enriched pathways included “2‐Oxocarboxylic acid metabolism” (15.364), “Fat digestion and absorption” (12.505), “TCA cycle” (12.003), “Biosynthesis of unsaturated fatty acids” (11.297), “Glycerolipid metabolism” (10.974), “Fatty acid elongation” (10.596), “Fatty acid metabolism” (9.912), “Biosynthesis of amino acids” (7.879), and “Cholesterol metabolism” (7.682). These results indicate that JMJD1A, functioning in an iron‐dependent manner, specifically controls gene networks implicated in adipogenesis and cellular energy metabolism.

### 2.5 Iron‐Dependent Regulation of H3K9me2 Demethylation at Target Loci

To evaluate whether JMJD1A regulates gene expression through iron‐dependent H3K9me2 demethylation at its target loci, we investigated H3K9me2 levels from the TSS to the transcription end site (TES) ±0.5 kb of the 291 JMJD1A target genes identified earlier and compared these levels to the genome‐wide average (Figure 3D). First, we analyzed **JMJD1A‐KD** (sh‐Jmjd1a) cells and control (sh‐Empty) cells 2 days after the induction of differentiation. At these 291 target loci, the relative H3K9me2 levels in JMJD1A‐KD cells compared to control cells were higher than the genome‐wide average (Figure 3D, top panel). These findings suggest that endogenous JMJD1A selectively demethylates H3K9me2 at genes crucial for adipogenesis, particularly those involved in the PPAR signaling pathway and other energy metabolism processes, during differentiation. Next, to verify the iron dependency of JMJD1A‐mediated H3K9me2 demethylation, we compared JMJD1A‐KD cells expressing JMJD1A(WT) with those expressing an iron‐coordination‐site defective mutant, JMJD1A(H1122A), on day 2 of differentiation. JMJD1A‐KD cells expressing JMJD1A(WT) demonstrated reduced H3K9me2 levels at JMJD1A target loci compared with JMJD1A‐KD cells expressing the control LacZ at these same target loci, relative to the genome‐wide average (Figure 3D, upper middle panel). Conversely, cells expressing JMJD1A(H1122A) showed higher H3K9me2 levels compared with JMJD1A(WT)‐expressing cells at these same target loci, relative to the genome‐wide average (Figure 3D, lower middle panel). These observations strongly support the conclusion that JMJD1A mediates H3K9me2 demethylation in an iron‐dependent manner. Additionally, treating differentiating cells with the iron chelator deferoxamine (DFO, 100 μM) during the first 2 days of induction resulted in persistently increased H3K9me2 levels compared to vehicle‐treated cells at these JMJD1A target loci, relative to the genome‐wide average, on day 2 of differentiation based on analysis of previously reported data, GSE174136 (Suzuki et al. 2023) (Figure 3D, bottom panel). This finding further underscores that the demethylase activity of JMJD1A at its target loci during adipocyte differentiation highly depends on iron availability.

### 2.6 Genome‐Wide Assessment of TSS‐Proximal H3K9me2 Demethylation by JMJD1A

To investigate how JMJD1A regulates H3K9me2 demethylation around TSSs on a genome‐wide scale, we analyzed H3K9me2 levels within ±1.5 kb of each TSS on day 2 of differentiation. Comparisons were made between (i) JMJD1A‐KD (sh‐Jmjd1a) cells and control (sh‐Empty) cells, (ii) JMJD1A‐KD cells expressing JMJD1A(WT) and JMJD1A‐KD cells expressing control LacZ, and (iii) JMJD1A‐KD cells expressing JMJD1A(H1122A) and JMJD1A‐KD cells expressing JMJD1A(WT) (Figure 3E). Based on these comparisons, TSS regions were categorized into three clusters. Cluster A1, encompassing 7487 TSSs, showed a pronounced trend toward H3K9me2 demethylation by JMJD1A(WT) in JMJD1A‐KD cells, but not by expression of the iron‐coordination‐site defective mutant JMJD1A(H1122A) in JMJD1A‐KD cells (Figure 3E, top panel). In contrast, Cluster A3, comprising 5330 TSSs, showed a pattern opposite to that of Cluster A1 (Figure 3E, bottom panel). Notably, Cluster A2, which includes 18,322 TSSs, showed minimal changes in H3K9me2 levels across all conditions (Figure 3E, middle panel). Subsequent KEGG pathway analysis (Figure 3E, right panels) indicated that Cluster A1 includes genes linked to “alpha‐Linolenic acid metabolism”, “Fat digestion and absorption,” “Glycerophospholipid metabolism”, and the “PPAR signaling pathway” (Figure 3E, top right panel). Compared with cluster A2, which had the smallest shift in H3K9me2, cluster A1 indicates that genes that are iron‐dependently demethylated via JMJD1A are closely associated with adipocyte differentiation. Cluster A3, on the other hand, featured only three enriched pathways at FDR < 0.01, none of which were strongly associated with adipogenesis (Figure 3E, bottom right panel). These findings suggest that the majority of TSS‐proximal loci are not significantly affected by JMJD1A during adipocyte differentiation, indicating that JMJD1A specifically targets regions of genes associated with adipogenesis. Taken together, our integrated RNA‐seq and CUT&Tag analyses results suggest that JMJD1A is essential for adipocyte differentiation, acting as an iron‐dependent H3K9me2 demethylase to drive the transcription of crucial adipogenesis‐associated genes. By combining both datasets, we identified 133 genes that appear to be regulated through H3K9me2 demethylation in an iron‐dependent manner by JMJD1A during adipogenesis (Figure 3F, top panel and Figure S2). These genes are primarily enriched in pathways such as “Fat digestion and absorption” and “PPAR signaling” (Figure 3F, bottom panel). These findings emphasize the central role of JMJD1A in coordinating PPAR‐driven and metabolic gene networks during adipocyte differentiation.

### 2.7 At the Megabase Scale, JMJD1A‐Mediated H3K9me2 Demethylation Aligns With A Compartments and Regions With Low Lamin B1 Levels

**FIGURE 4:**
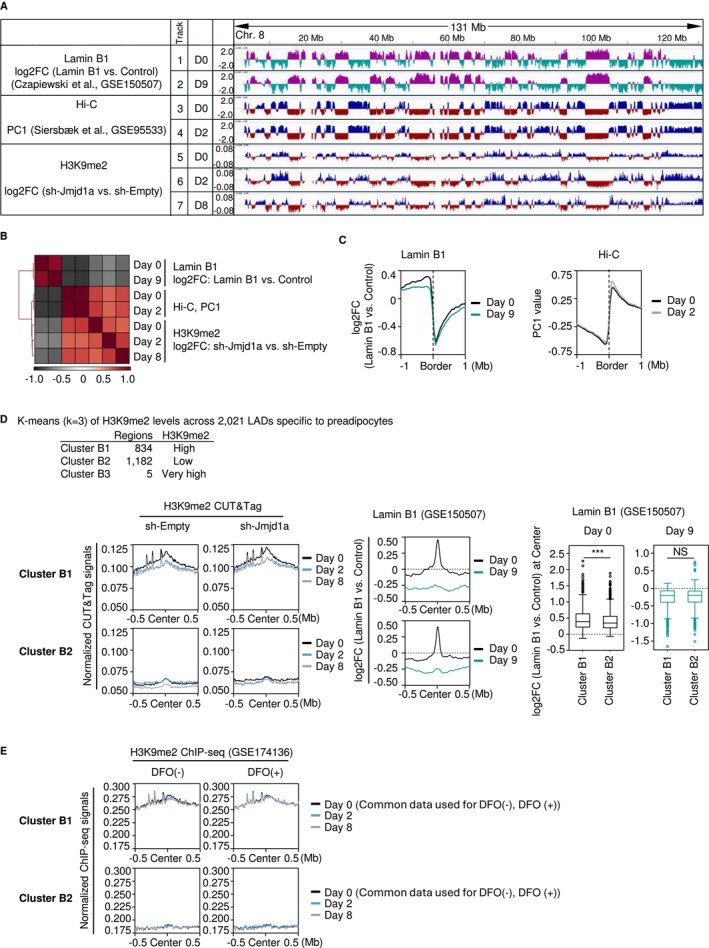
Subset‐Dependent H3K9me2 Demethylation in LAD Regions. (A) Genome browser overview of histone modification changes in LAD regions and their association with A/B compartments. Public lamin B1 DamID data (GSE150507) on days 0 and 9 of 3 T3‐L1 differentiation are shown in Tracks 1 and 2 as log2FC relative to the DamID control. Tracks 3 and 4 show published Hi‐C PC1 values (GSE95533) on days 0 and 2. Tracks 5 to 7 illustrate H3K9me2 log2FC values (JMJD1A‐KD [sh‐Jmjd1a] vs. control [sh‐Empty]) on days 0, 2, and 8 of differentiation, based on CUT&Tag data. All tracks correspond to chromosome 8. D0, D2, D8, and D9 indicate days 0, 2, 8, and 9 of differentiation, respectively. (B) Pearson correlation matrix of lamin B1 DamID, Hi‐C, and H3K9me2 CUT&Tag datasets. The matrix was calculated across the genome in 100‐kb bins, including DamID data on days 0 and 9, Hi‐C data on days 0 and 2, and H3K9me2 data on days 0, 2, and 8. (C) Aggregation plots at the A/B compartment boundaries. Lamin B1 DamID log2FC values on days 0 and 9 (left panel) and Hi‐C PC1 log2FC values on days 0 and 2 (right panel) are shown within ±1 Mb of the compartment boundaries, identified on day 2 of differentiation. The right panel displays the same data as the top panel of Figure 1C. (D) Clustering of 2021 LADs specific to preadipocytes (day 0 vs. day 9; GSE150507) based on H3K9me2 CUT&Tag signals in control (sh‐Empty) and JMJD1A‐KD (sh‐Jmjd1a) cells on days 0, 2, and 8. Five outlier regions with markedly increased H3K9me2 signals were classified as Cluster B3. The remaining 2016 regions were divided into Cluster B1 (834 regions, relatively high H3K9me2) and Cluster B2 (1182 regions, relatively low H3K9me2). Aggregation plots of normalized H3K9me2 signals in control (sh‐Empty) and JMJD1A‐KD (sh‐Jmjd1a) cells are shown within ±0.5 Mb of the center of LADs specific to preadipocytes for both clusters (left panels). Lamin B1 levels (log2FC vs. DamID control) at the center of undifferentiated‐specific LADs on days 0 and 9 in Clusters B1 and B2 are shown in the middle panels, and box plots in the right panels compare lamin B1 levels at the center in Clusters B1 and B2 on days 0 and 9. Statistical significance was evaluated using the Mann–Whitney U test. Box plots show the median, quartiles, whiskers representing the minimum and maximum, and open circles indicating outliers. ***p < 0.005; NS, no significant difference. (E) Effect of DFO on H3K9me2 in Clusters B1 and B2. 3 T3‐L1 cells were treated with DFO (100 μM) or vehicle for 2 days starting at the onset of differentiation, and H3K9me2 ChIP‐seq signals (GSE174136) in both groups were assessed on days 0, 2, and 8. Aggregation plots of normalized H3K9me2 signals for Clusters B1 and B2 are shown. The day 0 data for both treatments were derived from the same dataset.

LADs typically demonstrate an inverse correlation with A compartments. Considering our previous finding that JMJD1A‐mediated H3K9me2 demethylation during adipocyte differentiation overlaps with A compartments at the megabase scale, it is supposed that such demethylation would coincide with genomic regions characterized by low lamin B1 enrichment. To test this hypothesis, we compared our H3K9me2 CUT&Tag dataset and Hi‐C dataset with publicly available datasets of DNA adenine methyltransferase identification (DamID) for lamin B1 (lamin B1 DamID) from undifferentiated (day 0) and differentiated (day 9) 3T3‐L1 cells (Czapiewski et al. 2022). We found that endogenous JMJD1A‐mediated H3K9me2 demethylation (i.e., higher H3K9me2 levels in JMJD1A‐KD [sh‐Jmjd1a] cells compared with control [sh‐Empty] cells) indeed localizes specifically to A compartments and regions characterized by low lamin B1 levels (Figure 4A).

From a genome‐wide perspective, the extent of endogenous JMJD1A‐mediated H3K9me2 demethylation positively correlated with PC1 values derived from Hi‐C data, and negatively correlated with lamin B1 enrichment (Figure 4B). Furthermore, lamin B1 levels transitioned from high to low, shifting from positive to negative values at A/B compartment boundaries, where a corresponding shift from the B to the A compartment was observed (Figure 4C). Taken together, these findings reinforce our earlier conclusion that JMJD1A predominantly demethylates H3K9me2 within the A compartment, thereby contributing to transcriptional regulation in regions characterized by low lamin B1 enrichment levels.

### 2.8 H3K9me2 Changes During LAD Reorganization

Our data indicate that JMJD1A‐mediated H3K9me2 demethylation predominantly occurs within A compartments, which largely overlap with domains exhibiting low levels of lamin B1. However, this does not entirely rule out the possibility that H3K9me2 plays a specific role within LADs during adipocyte differentiation. Rather, H3K9me2 may function within LADs in a distinct manner during adipocyte differentiation, as supported by several lines of evidence. First, H3K9me2 is generally enriched in LADs, and its levels correlate well with lamin B1 levels (Poleshko et al. 2017). Indeed, artificial recruitment of the H3K9me2 methyltransferase G9a to specific genomic loci has been shown to promote LAD reorganization in an enzyme‐dependent manner in HEK293 cells (See et al. 2020). Contrarily, LADs and A/B compartments show no substantial changes during differentiation in 3T3‐L1 cells (Figure 4A), suggesting that higher‐order chromatin organization at the megabase scale is largely maintained throughout the terminal differentiation of adipocytes. Nevertheless, recent studies have demonstrated that a very limited subset of LADs undergoes reorganization during adipocyte differentiation in 3T3‐L1 cells (Czapiewski et al. 2022). These findings suggest that H3K9me2 may influence the dynamic remodeling of a specific subset of LADs during adipocyte differentiation. However, the underlying mechanisms and its relationship with JMJD1A‐KD or DFO treatment remain unclear. Therefore, we focused on 2021 LADs specific to preadipocytes, defined as genomic regions exhibiting reduced lamin B1 association on day 9 compared to day 0 of adipocyte differentiation, based on publicly available lamin B1 DamID data (Czapiewski et al. 2022). We then examined changes in H3K9me2 levels within these regions, as well as the effects of JMJD1A‐KD and DFO treatment. The 2021 LADs specific to preadipocytes were clustered based on H3K9me2 levels in JMJD1A‐KD (sh‐Jmjd1a) and control (sh‐Empty) cells on days 0, 2, and 8. The elbow method indicated that three clusters were an appropriate solution (Figure S3A). K‐means clustering identified a small set of five LADs with very high H3K9me2 (Cluster B3), whereas the remaining 2016 LADs were divided into Cluster B1 (relatively high H3K9me2) and Cluster B2 (comparatively low H3K9me2) (Figure 4D, left panel and S2B). In Cluster B1, H3K9me2 levels were highest at the LAD centers and gradually tapered toward ±0.5 Mb from the center (Figure 4D, left panel and Figure S3C). Additionally, H3K9me2 levels in Cluster B1 were markedly higher than those in cluster B2. This trend was observed not only in CUT&Tag analyses for H3K9me2 using JMJD1A‐KD (sh‐Jmjd1a) and control (sh‐Empty) cells, but also in chromatin immunoprecipitation followed by high‐throughput sequencing (ChIP‐seq) analyses of H3K9me2 using 3T3‐L1 cells treated with DFO (100 μM) or vehicle for 2 days after induction of adipocyte differentiation (GSE174136, (Suzuki et al. 2023)) (Figure 4E). Thus, these findings suggest that this trend is specific to the process of adipocyte differentiation.

A comparative analysis of lamin B1 levels at the centers of LADs specific to preadipocytes using aggregation plots revealed that Cluster B1 exhibited slightly, yet significantly, higher lamin B1 levels than Cluster B2 on day 0 (Figure 4D, middle and right panels). This finding aligns with the general notion that H3K9me2 enrichment and lamin B1 levels are closely correlated (Czapiewski et al. 2022; See et al. 2020; Padeken et al. 2022). However, notably, no significant difference in lamin B1 levels at the centers of LADs specific to preadipocytes was observed between Clusters B1 and B2 on day 9 (Figure 4D, middle and right panels), despite the substantial difference in H3K9me2 levels at that time (Figure 4D, left panel). Furthermore, aggregation plots of lamin B1 changes over time demonstrated a pronounced decrease in Lamin B1 from day 0 to day 9 in both Cluster B1 and Cluster B2, regions that show high and low H3K9me2 levels, respectively (Figure 4D, middle panel). Collectively, these findings suggest that the disassembly of LADs specific to preadipocytes during adipocyte differentiation occurred largely independently of the H3K9me2 state.

## 3 Discussion

In this study, we identified genomic regions that undergo JMJD1A‐mediated H3K9me2 demethylation through its iron‐dependent enzymatic activity during adipocyte differentiation. Notably, our findings suggest that JMJD1A operates differentially in the A and B compartments. Specifically, at the megabase scale, JMJD1A‐catalyzed, iron‐dependent H3K9me2 demethylation predominantly occurs in the A compartment. However, the underlying molecular mechanism remains unclear. One potential explanation is the genomic distribution of its target regions. In general, the A compartment exhibits a higher gene density than the B compartment (Gibcus and Dekker 2013). Therefore, the regulation of H3K9me2 by JMJD1A may appear more extensive within the A compartment. Another possible explanation involves phase separation, which is linked to large‐scale chromatin structural changes. Several JmjC domain‐containing histone lysine demethylases (JmjC‐KDMs) have been reported to participate in the spatiotemporal regulation of nuclear organization through phase separation (Li et al. 2022; Vicioso‐Mantis et al. 2022). Moreover, phosphorylation of JmjC‐KDMs is proposed to modulate molecular interactions between proteins and nucleic acids by altering their negative charges, thereby influencing protein phase separation (Karakatsanis et al. 2024). Given that JMJD1A phosphorylation has been observed during beige adipocyte differentiation (Abe et al. 2018) and in association with heat production in brown adipocytes (Abe et al. 2015), it is plausible that JMJD1A also undergoes phosphorylation during white adipocyte differentiation and participates in phase separation. Consequently, JMJD1A may actively mediate and/or recognize spatial genome structures.

H3K9me2 demethylation within the A compartment has been linked to selective and dynamic gene repression (Smith et al. 2021; Padeken et al. 2022). Building on this, we investigated whether JMJD1A regulates transcription through H3K9me2 demethylation in the A compartment and conducted detailed analyses of potential JMJD1A target genes. These analyses identified a gene set closely associated with PPAR signaling, which is a pathway crucial for adipocyte differentiation and energy metabolism, including lipid metabolism. Previous studies have shown that H3K9me2 loss triggers the cell type–specific upregulation of distinct gene subsets, which requires unique transcription factors (Methot et al. 2021; Padeken et al. 2022). Consistently, JMJD1A‐dependent regulation of *Pparg* expression, along with genes associated with the PPAR signaling pathway, suggests that the transcription factor PPARγ, once induced through H3K9 demethylation, may further regulate genes within newly demethylated regions. Collectively, these findings highlight that JMJD1A plays a crucial role in regulating cell type‐specific gene expression during adipocyte differentiation, not only by inducing *Pparg* expression but also by facilitating transcriptional changes within newly demethylated chromatin regions. In these regions, expressed PPARγ may collaborate with structurally relaxed regions to amplify or sustain the expression of adipocyte differentiation‐associated genes, ultimately supporting a model in which JMJD1A‐mediated chromatin remodeling integrates with transcription factor activity to establish cell‐type‐specific expression programs during differentiation.

In addition to the adipocyte differentiation‐associated genes regulated by JMJD1A, megabase scale observations have revealed that many other genes within the same compartment exhibit changes in H3K9me2. However, the extent to which the expression of these genes is specifically regulated by H3K9me2 remains unclear. Given that transcriptional regulation during cell differentiation is orchestrated through the interplay of transcriptional factors and chromatin structural changes, both the changes in H3K9me2 and the assembly of the transcriptional machinery, particularly transcriptional factors, may contribute to determining the specificity of the expression of these genes.

In addition to the role of JMJD1A in H3K9 demethylation within the A compartment, we identified an association between H3K9me2 demethylation and LADs, which generally align with the B compartment. H3K9me2 is known to be enriched in LADs, particularly in the nuclear periphery, where it is often associated with transcriptional repression (Padeken et al. 2022). Our findings indicate that in regions where LADs specifically decrease during adipocyte differentiation (namely, LADs specific to preadipocytes), the notable differences in H3K9me2 levels between regions with relatively high H3K9me2 (Cluster B1) and those with relatively low H3K9me2 (Cluster B2) appear to correspond with limited but discernible differences in lamin B1 levels at the central regions of these LADs (Figure 4D, right panel). This observation suggests that H3K9me2 may partially influence LAD dynamics. Nonetheless, lamin B1 levels declined during differentiation regardless of the initial H3K9me2 status, indicating that LAD degradation occurs largely independently of baseline H3K9me2 levels. However, this does not exclude the possibility that, rather than their initial levels, even limited dynamic changes in H3K9me2 within individual LADs may still contribute to LAD reorganization during adipocyte differentiation, although further studies are required to test this hypothesis.

From the perspective of histone H3K9 methyltransferases (HMTs), recent studies have demonstrated that H3K9me2 levels are regulated by multiple HMTs in a compartment‐specific manner within the A and B compartments. Specifically, in ES cells and differentiated mammalian cells, H3K9me2 in the A compartment is predominantly regulated by SETDB1, G9A, and GLP, whereas in the B compartment, all five major HMTs (G9A, GLP, SETDB1, SUV39H1, and SUV39H2) are involved (Fukuda et al. 2021; Padeken et al. 2022). In contrast, much less is known about how H3K9me2 demethylases function within these distinct compartments, particularly during adipocyte differentiation. Our findings provide new insights by demonstrating that JMJD1A contributes to H3K9me2 demethylation in the A compartment. However, we did not establish direct causal links between the experimental manipulation of JMJD1A expression or its iron‐dependent enzymatic activity and changes in compartment composition or LAD distribution, highlighting the need for more in‐depth studies to clarify these associations in the future. Additionally, several other enzymes, including JMJD2A, JMJD2B, JMJD2C, JMJD2D, jumonji C (JmjC) domain‐containing histone demethylase 1D (JHDM1D), PHD finger protein 2 (PHF2), and PHD finger protein 8 (PHF8), have been reported to demethylate H3K9me2 (Kooistra and Helin 2012). Among these, we previously demonstrated that JMJD2B, PHF2, and PHF8 regulate *Pparg* expression in an iron‐dependent manner during adipocyte differentiation, albeit with a lower contribution compared with JMJD1A (Suzuki et al. 2023). This suggests that multiple demethylases, in addition to JMJD1A, cooperate in this process. Future studies are needed to elucidate the specific contributions of these enzymes and their potential interplay in chromatin remodeling and transcriptional regulation.

Previous studies have demonstrated that JMJD1A regulates thermogenic adipocyte differentiation and function through a dual mechanism involving both its enzymatic H3K9me2 demethylation activity and its role in forming chromatin remodeling complexes with other proteins (Abe et al. 2015; Abe et al. 2018). Similarly, an isoform of the histone demethylase F‐box and leucine‐rich repeat protein (FBXL10, also known as KDM2B), which lacks a JmjC domain, has been shown to regulate white adipocyte differentiation through a non‐enzymatic mechanism that facilitates the formation of polycomb repressive complex 1 (Inagaki et al. 2015). These findings highlight the functional versatility of histone demethylases, which can regulate cellular processes either through their catalytic demethylation activities or through enzymatic‐activity–independent recruitment of chromatin complexes. In this study, we used KD strategies combined with rescue experiments using WT JMJD1A and an iron‐coordination‐defective JMJD1A mutant to identify target genes regulated by JMJD1A‐dependent H3K9me2 demethylation. Our findings indicate the crucial role of the enzymatic activity of JMJD1A in controlling adipocyte differentiation.

These results pave the way for deeper investigations into how JMJD1A interacts with other regulatory factors to modulate chromatin architecture and transcriptional outcomes during adipogenesis. Understanding these mechanisms will provide valuable insights into the complex interplay between the enzymatic and nonenzymatic functions of histone demethylases in the regulation of cellular differentiation.

## 4 Experimental Procedures

### 4.1 Cell Culture

All cell lines used in this study were generated as previously described (Suzuki et al. 2023). Briefly, JMJD1A‐KD (sh‐Jmjd1a) and control (sh‐Empty) cell lines were established by stably expressing short hairpin RNA (shRNA) targeting *Jmjd1a* (sh‐Jmjd1a) or control shRNA (sh‐Empty) in 3T3‐L1 preadipocytes (provided by Dr. Howard Green) through retroviral infection, following a previously reported protocol (Suzuki et al. 2023). Retroviral expression vectors were packaged into infectious retroviral particles and used to infect 3T3‐L1 cells according to a protocol provided by Dr. Toshio Kitamura. Infected cells were selected using 10 μg/mL puromycin (Thermo Fisher, ANT‐PR‐1) or 10 μg/mL blasticidin S (Thermo Fisher, ANT‐BL‐1) to establish stable cell lines. For functional restoration experiments, a silent mutation conferring resistance to shRNA was introduced into the JMJD1A (WT) coding sequence to create a retroviral expression vector. An H1122A substitution was also introduced to generate a mutant vector encoding a JMJD1A protein with defective iron coordination. These plasmids, along with a control plasmid encoding LacZ, were used to produce retroviruses. The retroviral particles were then used to infect JMJD1A‐KD (sh‐Jmjd1a) cells, and stable cell lines were established through antibiotic selection. 3T3‐L1 cells and stable 3T3‐L1 lines generated as described above were cultured in Dulbecco's modified Eagle's medium–high glucose (DMEM; FUJIFILM Wako, 043–30,085) supplemented with 10% calf serum (CS) under 5% CO_2_ at 37°C. Two days after the cells reached confluence (designated as day 0 of differentiation induction), the culture medium was replaced with DMEM containing 10% fetal bovine serum (DMEM‐FBS), supplemented with 1.67 μM insulin (Sigma, I5500), 1 μM dexamethasone (Sigma, D4902), and 115 μg/mL 1‐isobutyl‐3‐methylxanthine (Sigma, I5879). After 48 h, the medium was replaced with DMEM‐FBS supplemented with 1.67 μM insulin alone, and cells were further incubated for an additional 48 h. The medium was subsequently replaced with fresh DMEM‐FBS every 2 days until day 8 of differentiation. For ChIP‐seq analysis using the anti‐H3K9me2 antibody [previously reported in (Suzuki et al. 2023), GSE174136], DFO (Sigma, D9533) was freshly diluted in DMEM (vehicle) and added to the culture medium at a final concentration of 100 μM at the start of adipocyte differentiation. DFO treatment was carried out for 48 h following the induction of adipocyte differentiation.

### 4.2 Purification of Recombinant Protein A–Tn5 (pA‐Tn5) and Loading of Mosaic‐End Oligonucleotides

The Protein A–Tn5 (pA‐Tn5) fusion enzyme was purified following protocols.io (dx.doi.org/10.17504/protocols.io.8yrhxv6) and previously described methods (Kaya‐Okur et al. 2019; Kaya‐Okur et al. 2020), with minor modifications. Briefly, the plasmid 3XFlag‐pA‐Tn5‐FI (Addgene, 124,601) was transformed into T7 Express lysY/Iq competent *E. coli* (NEB, C3013I), and transformants were grown overnight on LB agar plates containing 100 μg/mL ampicillin. A single colony was transferred to LB broth supplemented with ampicillin and cultured at 37°C until the OD600 reached approximately 0.6. The cultures were cooled on ice for 30 min, induced with 0.25 mM IPTG, and incubated on a shaker at 18°C for 20 h. Cells were harvested by centrifugation at 4°C, flash‐frozen in liquid nitrogen, and stored at −80°C.

Cell pellets from a 100 mL culture were resuspended in 10 mL of HEGX buffer (20 mM HEPES‐KOH, pH 7.2, 0.8 M NaCl, 1 mM EDTA, 10% glycerol, 0.2% Triton X‐100, 1 mM PMSF, 2 μg/mL pepstatin A) and sonicated using a Branson Sonifier 150 for 10 cycles of 30 s ON/30 s OFF at 70% power. Following sonication, the sample was centrifuged, and the resulting supernatant was treated by adding polyethylenimine (PEI, Sigma, P3143; pH 7.1) to a final concentration of 0.26% at 4°C with gentle rotation to remove E. coli DNA. After a second centrifugation to eliminate insoluble residues, the clarified supernatant was incubated with pre‐washed chitin resin (NEB, S6651S) at 4°C for 2 h. The resin was packed into a column (Bio‐Rad, 7,371,517) and extensively washed with HEGX buffer. The pA‐Tn5 enzyme was cleaved from the intein tag by incubating the resin in HEGX buffer containing 100 mM DTT for 36 h at 4°C. Fractions with the highest protein concentrations were pooled and dialyzed twice using dialysis tubing (Spectrum Labs, 132,118) against 2× dialysis buffer (100 mM HEPES‐KOH, pH 7.2, 0.2 M NaCl, 0.2 mM EDTA, 2 mM DTT, 0.2% Triton X‐100, 20% glycerol). Protein concentration was determined by measuring absorbance at 280 nm (A280) using molecular weight (74,796.37) and extinction coefficient (93,975) values predicted by ProtParam (https://web.expasy.org/protparam/). The purified pA‐Tn5 enzyme (11.3 μM) was stored at −20°C in 40% glycerol.

To load mosaic‐end oligonucleotides, Tn5ME‐rev (5′‐[phos]CTGTCTCTTATACACATCT‐3′) was annealed to either Tn5ME‐A (5´‐TCGTCGGCAGCGTCAGATGTGTATAAGAGACAG‐3′) or Tn5ME‐B (5´‐GTCTCGTGGGCTCGGAGATGTGTATAAGAGACAG‐3′) at a final concentration of 100 μM each (200 μM total). The two annealed solutions were combined and incubated with pA‐Tn5 at a final concentration of 10.1 μM for 1 h at 23°C to form pA‐Tn5–adapter complexes. These complexes were diluted to 45 nM immediately before use.

### 4.3 Tn5 Transposase‐Based Cleavage Under Targets & Tagmentation (CUT&tag)

CUT&Tag was performed following the bench‐top Cut&Tag V.3 protocol available on protocols.io (dx.doi.org/10.17504/protocols.io.bcuhiwt6) (Kaya‐Okur et al. 2019). Concanavalin A (ConA) beads (Bangs Labs, BP531) were washed twice with Binding Buffer (20 mM HEPES‐KOH, pH 7.9, 10 mM KCl, 1 mM CaCl_2_, 1 mM MnCl_2_) and resuspended in 10 μL of buffer per sample. For nuclear fraction preparation, cells cultured in 10 cm dishes were washed with PBS, trypsinized using 0.05% trypsin, collected, washed with PBS, resuspended in ice‐cold NE1 Buffer (20 mM HEPES‐KOH, pH 7.9, 10 mM KCl, 0.5 mM spermidine, 0.1% Triton X‐100, 20% glycerol), incubated on ice for 10 min, and centrifuged to isolate nuclei. The nuclei were then resuspended in Wash Buffer (20 mM HEPES‐NaOH, pH 7.5, 150 mM NaCl, 0.5 mM spermidine) and adjusted to 1 × 10^6^ nuclei/mL. A total of 100 μL of the nuclei suspension (1 × 10^5^ nuclei) was conjugated with 10 μL of ConA beads at RT for 5 min. After removing the supernatant using a magnetic stand, 50 μL of primary antibody solution (10 μg/mL in Antibody Buffer: 20 mM HEPES‐NaOH, pH 7.5, 150 mM NaCl, 0.5 mM spermidine, 2 mM EDTA, 0.1% BSA, and protease inhibitor cocktail) was added. The nuclei‐bead complexes were incubated overnight at 4°C with gentle rocking using either mouse monoclonal anti‐H3K9me2 antibody (provided by Dr. Hiroshi Kimura) or anti‐H3K27me3 antibody (Millipore, 07–449). After overnight incubation, the beads were washed with Wash Buffer and incubated at RT for 30 min in Wash Buffer containing protease inhibitors and 10 μg/mL secondary antibody (anti‐mouse IgG or anti‐rabbit IgG; Rockland, 611–201‐122). The beads were then washed again with Wash Buffer, followed by incubation at RT for 30 min with 45 nM pA‐Tn5‐adapter complex in 300‐Wash Buffer (20 mM HEPES‐NaOH, pH 7.5, 300 mM NaCl, 0.5 mM spermidine, protease inhibitors) under gentle mixing. After additional washes with 300‐Wash Buffer, tagmentation was performed by adding 100 μL of Tagmentation Buffer (20 mM HEPES‐NaOH, pH 7.5, 300 mM NaCl, 0.5 mM spermidine, 10 mM MgCl_2_) and incubating at 37°C for 1 h. DNA was extracted using the DNA Clean & Concentrator‐5 kit (Zymo Research, D4014) and eluted in 25 μL of nuclease‐free water. Library preparation was performed using PCR with 21 μL of extracted DNA, 2 μL each of i5 and i7 primers (10 μM each; sequences referenced from Illumina Adapter Sequences, Illumina, 1,000,000,002,694 v19), and 25 μL of High‐Fidelity 2X PCR Master Mix (NEB, M0541S). The PCR conditions were as follows: an initial incubation at 72°C for 5 min, followed by 98°C for 30 s, 13 cycles of 98°C for 10 s and 63°C for 10 s, and a final extension at 72°C for 1 min. PCR products were purified using AMPure XP beads (Beckman, A63880) by mixing 45 μL of PCR product with 90 μL of beads (2:1 ratio). The supernatant was removed using a magnetic stand, and the beads were washed with 80% ethanol. DNA was eluted, and library concentrations were assessed using the TapeStation 4150 system with High Sensitivity D5000 ScreenTape (Agilent, 5067–5592) and Qubit dsDNA HS assays (Invitrogen, Q32851). Libraries were pooled in equimolar amounts and subjected to double size selection using AMPure XP beads to remove adapter dimers (1.3:1 bead‐to‐sample ratio). Sequencing was performed on a NovaSeq 6000 or NovaSeq X platform (Illumina) for paired‐end 151 cycles. The dataset has been deposited in the DNA Data Bank of Japan (DDBJ) under the accession number DRA015768/PRJDB15347 (for analyses using JMJD1A‐KD (sh‐Jmjd1A) cells and control (sh‐Empty) cells) and in the NCBI Gene Expression Omnibus (GEO) under the accession number GSE284471 (for analyses using LacZ‐expressing JMJD1A‐KD cells, JMJD1A(WT)‐expressing JMJD1A‐KD cells, and JMJD1A(H1122A)‐expressing JMJD1A‐KD cells).

### 4.4 CUT&Tag Data Analysis

Bioinformatic analyses were performed using the indicated tools with default settings unless otherwise noted. Paired‐end reads were pre‐processed using fastp (version 0.21.0 for DRA015768/PRJDB15347 or Galaxy Version 0.24.0 + galaxy3 for GSE284471) and mapped to the mouse mm9 reference genome using Bowtie2 (‐very‐sensitive, version 2.4.5 for DRA015768/PRJDB15347 or Galaxy Version 2.5.3 + galaxy1 for GSE284471). Mitochondrial DNA reads were removed from the resulting BAM files using Samtools (version 1.11) with the grep ‐v chrM option for DRA015768/PRJDB15347, or Samtools view (‐e ‘rname! = “chrM”’, version 1.20) for GSE284471. Reads with a mapping quality of 40 or higher (–F 0 × 4–q 40) were retained. Duplicate reads were removed using Samtools fixmate (−m) and markdup (−r). The resulting deduplicated BAM files were used to generate bigWig files with the bamCoverage function of deepTools (−e, version 3.5.0 for DRA015768/PRJDB15347 or Galaxy Version 3.5.4 + galaxy0 for GSE284471), with normalization applied using Counts Per Million (CPM; normalizeUsing CPM).

To combine replicates for each condition, bigWig files were merged with bigwigAverage in deepTools (Galaxy Version 3.5.4 + galaxy0), and the resulting coverage data were visualized in IGV (Integrative Genomics Viewer, version 2.8.7). Principal component analysis (PCA) plots were generated from individual replicate data with multiBigwigSummary and plotPCA in deepTools (Galaxy Version 3.5.4 + galaxy0). To calculate log2FC values between two conditions, bigwigCompare of deepTools (Galaxy Version 3.5.4 + galaxy0) was used, optionally setting a specific bin size.

For H3K9me2 analyses at transcription start sites (TSSs), a bed file containing all TSS positions in the genome was generated from a GTF file obtained from the UCSC website (https://hgdownload.soe.ucsc.edu/goldenPath/mm9/bigZips/genes/). With this bed file, the functions computeMatrix and plotHeatmap in deepTools (Galaxy Version 3.5.4 + galaxy0) were applied to calculate and visualize H3K9me2 log2FC values within a ± 1.5 kb window around TSSs (reference‐point—referencePoint center—beforeRegionStartLength 1500—afterRegionStartLength 1500). The generation of heatmaps and k‐means clustering of log2FC values for H3K9me2 analysis at TSSs was performed using heatmap2 (Galaxy Version 3.2.0 + galaxy1) and Cluster 3.0 (http://bonsai.hgc.jp/~mdehoon/software/cluster/software.htm), respectively.

To examine H3K9me2 levels at gene bodies, the longest transcript for each gene was selected based on the same GTF file, and the corresponding regions from the TSS to the TES (transcription end site) were used to create a bed file. Aggregation plots were then generated with computeMatrix and plotProfile in deepTools (Galaxy Version 3.5.4 + galaxy0), using scale‐regions—regionBodyLength 5000—beforeRegionStartLength 500—afterRegionStartLength 500—binSize 500. Previously reported H3K9me2 ChIP‐seq datasets for 3 T3‐L1 cells with or without DFO treatment (GSE174136, (Suzuki et al. 2023)) were analyzed using the same approach.

### 4.5 RNA‐Seq

Cells were collected at the indicated time points before or after differentiation induction, and total RNA was isolated using the RNeasy Lipid Tissue Mini Kit (Qiagen, 74,804) according to the manufacturer's instructions, including DNase (Qiagen, 79,254) treatment. Library construction was performed with the NEBNext Poly(A) mRNA Magnetic Isolation Module and the NEBNext UltraTM II Directional RNA Library Prep Kit (New England Biolabs). Sequencing was carried out on a NovaSeq 6000 platform (Illumina) in a 150 × 2 paired‐end format by Rhelixa Inc. The resulting RNA‐seq datasets have been deposited in the GEO under the accession number GSE269147.

### 4.6 RNA‐Seq Data Analysis

After preprocessing reads with fastp (Galaxy Version 0.23.2 + galaxy0), the filtered reads were aligned to the mm9 mouse genome using HISAT2 (Galaxy Version 2.2.1 + galaxy1). Read counting was conducted with featureCounts (Galaxy Version 2.0.3 + galaxy2). Differentially expressed genes (DEGs) were identified using edgeR (Galaxy Version 3.36.0 + galaxy4) with a false discovery rate (FDR) cutoff of < 0.05. Gene ontology (GO) analysis was performed using ShinyGO 0.80 (http://bioinformatics.sdstate.edu/go/). Heatmaps of the expression levels of selected genes were produced based on TPM (transcripts per million) values using heatmap2 (Galaxy Version 3.1.3.1 + galaxy0).

### 4.7 Public Hi‐C Data Analysis

Processed Hi‐C data (Hi‐C Summary files) for 3 T3‐L1 differentiation were obtained from NCBI GEO (GSE95533) (Siersbæk et al. 2017). Replicates were merged into Tag Directories using makeTagDirectory of HOMER (version 4.11.1), and principal component 1 (PC1) values were calculated with runHiCpca.pl. The resulting bedGraph files were converted to bigWig format with “Convert BedGraph to BigWig” (Galaxy Version 1.0.1). Pearson's correlation coefficients were computed and displayed as a heatmap using the multiBigwigSummary (‐binSize 100,000) and plotCorrelation (‐corMethod pearson) functions of deepTools (Galaxy Version 3.5.4 + galaxy0). Boundaries between A and B compartments were identified by changes in the PC1 values from positive to negative or vice versa. Aggregation plots for compartment borders were generated with computeMatrix and plotProfile in deepTools (Galaxy Version 3.5.4 + galaxy0), employing reference‐point—referencePoint center—beforeRegionStartLength 1,000,000—afterRegionStartLength 1,000,000—binSize 50,000.

### 4.8 Public Lamin B1 DamID Data Analysis

Processed lamin B1 DamID data for 3 T3‐L1 cells were obtained from NCBI GEO (GSE150507) (Czapiewski et al. 2022). A bed file containing regions lamina‐associated predominantly on day 0 (LADs specific to preadipocytes) was also retrieved. To examine H3K9me2 CUT&Tag signals around these LADs, computeMatrix and plotProfile from deepTools (Galaxy Version 3.5.4 + galaxy0) were used, and k‐means clustering was applied to categorize LADs based on H3K9me2 levels (reference‐point—referencePoint center—beforeRegionStartLength 500,000—afterRegionStartLength 500,000—binSize 10,000—kmeans 3). The number of clusters was determined with the elbow method, and plots were generated using the KMeans function from scikit‐learn (v1.0.2). One cluster that displayed extraordinarily high H3K9me2 levels (more than 20‐fold higher than in other clusters; only five out of 2021 LADs specific to preadipocytes) was considered an outlier and removed from subsequent analysis.

### 4.9 Statistical Analysis

Statistical differences in Figure 4D were examined using Python (v. 1.8.0) by performing the Mann–Whitney U test to compare the central tendencies between two distributions. Data are presented as box‐and‐whisker plots. The box‐and‐whisker plots provide a statistical summary that includes the median, quartiles (boxes), range from the minimum to the maximum values (whiskers), and outliers (open circles). *p < 0.05 was considered statistically significant, while ***p < 0.005 indicated a highly significant difference.

## Author Contributions

**Shinnosuke Masuda:** methodology, investigation, formal analysis. **Tetsuro Komatsu:** formal analysis, data curation, funding acquisition. **Safiya Atia:** formal analysis, data curation, validation. **Tomohiro Suzuki:** investigation. **Mayuko Hayashi:** investigation. **Atsushi Toyoda:** resources. **Hiroshi Kimura:** resources. **Takeshi Inagaki:** conceptualization, investigation, funding acquisition, project administration, supervision, writing – original draft, writing – review and editing, visualization.

## Conflicts of Interest

The authors declare no conflicts of interest.

## Supporting information


Data S1.

## Data Availability

Next‐generation sequencing datasets reported in this study have been deposited in the NCBI Gene Expression Omnibus (GEO) under the accession numbers GSE269147 (RNA‐seq) and GSE284471 (CUT&Tag), and in the DNA Data Bank of Japan (DDBJ) under the accession number DRA015768/PRJDB15347 (CUT&Tag).
