# Supplementary material for: Iron‐Dependent JMJD1A‐Mediated Demethylation of H3K9me2 Regulates Gene Expression During Adipogenesis in a Spatial Genome Organization‐Dependent Manner
Source: Genes Cells. 2025 Apr 28;30(3):e70023. doi: 10.1111/gtc.70023 (PMC12035669; doi:10.1111/gtc.70023)
Supplement: Supplementary file 1 — Data S1. [file GTC-30-0-s001.pdf]

Figure S1

A

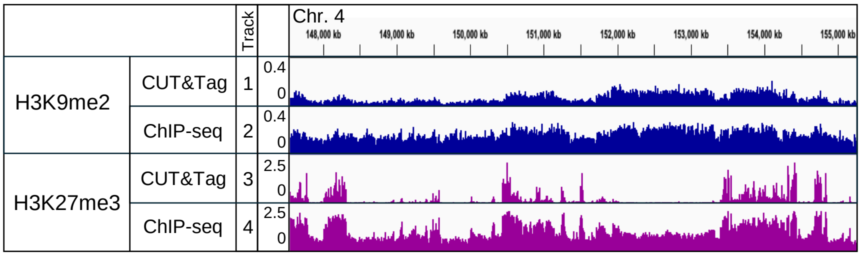

B

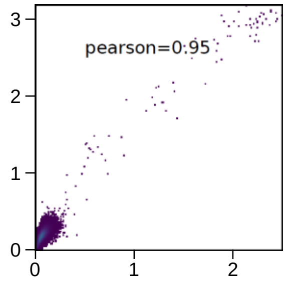

Figure S1. H3K9me2 profiles between CUT&Tag and ChIP-seq reveal a strong positive correlation
(A) IGV snapshot of the H3K9me2 and H3K27me3 profiles in sh-Empty 3T3-L1 cells (Day 0) obtained by CUT&Tag (DRA015768/PRJDB15347) and in 3T3-L1 cells (Day 0) obtained by ChIP-seq (GSE174136). **(B)** The Pearson correlation coefficient between the genome-wide H3K9me2 signals from CUT&Tag and ChIP-seq was 0.95.

Figure S2

A530016L24Rik	*AU015836*	*Acat2*	*Aco1*	*Actn3*	*Adcyap1r1*	*Adgrb2*	*Adipor2*
Agpat2	*Aifm2*	*Akap1*	*Aldh6a1*	*Amotl2*	*Angptl4*	*Aoc3*	*Apol6*
Aqp1	*Atp1a2*	*Bcat2*	*C3*	*Cabp4*	*Car5b*	*Cavin2*	*Cbr2*
Cers4	*Chd4*	*Chst1*	*Cidec*	*Cldn15*	*Cluh*	*Cmklr1*	*Col18a1*
Col5a3	*Crocc2*	*Cyb5b*	*Cyp51*	*Dgat2*	*Dhcr24*	*Disp2*	*Dlk1*
Dpep1	*Dusp18*	*Dusp2*	*Ecm1*	*Epb41l1*	*Fabp4*	*Fam117a*	*Fam83a*
Fdxr	*Fis1*	*Gpd1*	*Gramd1b*	*Hadha*	*Hipk2*	*Hsd11b1*	*Hsd17b12*
Il15ra	*Insig1*	*Kcnk3*	*Krt13*	*Lama5*	*Lcn2*	*Ldlr*	*Limk1*
Lipe	*Lpin1*	*Ltc4s*	*Mdfi*	*Mocs1*	*Msln*	*Mtss2*	*Mylk*
Ncapg2	*Ndrg2*	*Notch1*	*Nr1h3*	*Oaf*	*Ogdh*	*Pank3*	*Paqr4*
Pcx	*Pdpk1*	*Pitpnm2*	*Plaat3*	*Plin4*	*Plpp1*	*Pnpo*	*Pole4*
Pparg	*Ppp1r15b*	*Ppp2r1b*	*Psen2*	*Ptpn6*	*Ptprn*	*Qsox1*	*Rarres2*
Rassf2	*Rdh12*	*Reep6*	*Ren1*	*Retn*	*Rreb1*	*Scarb1*	*Scd1*
Sema3f	*Septin9*	*Sh2b2*	*Slc25a5*	*Slc43a3*	*Sncg*	*Sorbs1*	*Sphk1*
Spon2	*Spry4*	*Srebf1*	*Syn2*	*Syt13*	*Tgm2*	*Thbs2*	*Thrsp*
Timp4	*Tkt*	*Tmcc3*	*Tmem120a*	*Tnfaip2*	*Tnxb*	*Trf*	*Trim29*
Ttyh2	*Ucp2*	*Wfdc21*	*Wwox*	*Xbp1*			

Figure S2.
List of 133 genes putatively regulated by JMJD1A in an enzyme activity–dependent manner during adipogenesis.

Figure S3

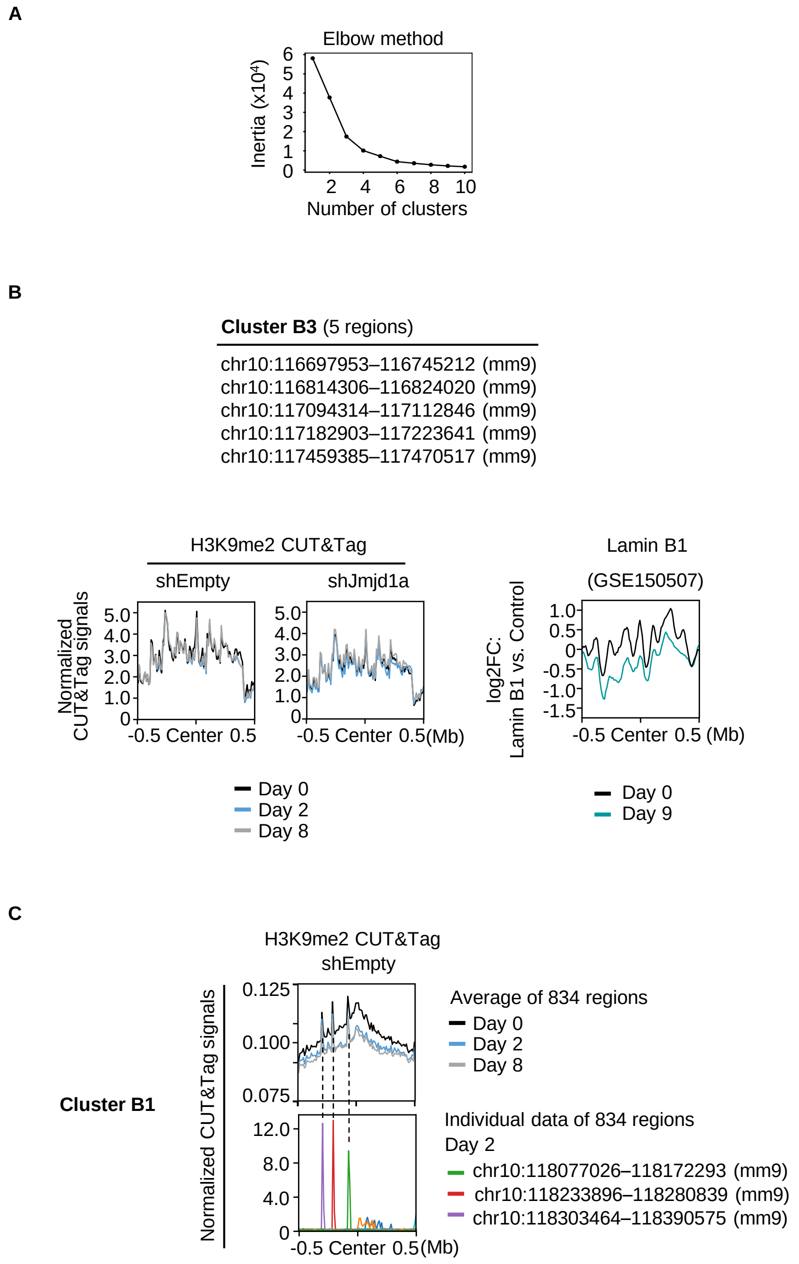

Figure S3.
(A) Determination of the optimal cluster number via the elbow method for H3K9me2 signals in 2,021 LADs specific to preadipocytes of JMJD1A-KD (sh-Jmjd1a) cells and control (sh-Empty) cells on days 0, 2, and 8.
(B) Detailed information regarding the five outlier regions classified as Cluster B3, including their genomic coordinates (top panel), and the aggregation plots of their normalized H3K9me2 signals in JMJD1A-KD (sh-Jmjd1a) cells and control (sh-Empty) cells (bottom left panels). The bottom right panel shows the aggregation plot of lamin B1 DamID signals (log2FC relative to control) on days 0 and 9 for these regions.
(C) Three nonspecific outliers in Cluster B1 are illustrated in sh-Empty cells to clarify the spike-like patterns in the left panel of Figure 4D.
